# "The Hospital" Nursing Mirror

**Published:** 1901-02-23

**Authors:** 


"The Hospital" Nursing Mirror.

Being the Nursing Section of "The Hospital."

[Contributions for this Section of "The Hospital" should be addressed to the Editor, "The Hospital" Nursing Mirror, 28 & 29 Southampton Street Strand, London, W.C.]

### Notes on News from the Nursing World.

### THE KING AND THE ROYAL RED CROSS.

His Majesty the King has conferred the decoration of the Royal Red Cross upon Miss Marian Lambert, of the Church of England Mission, who was matron of the temporary International Hospital, Peking, during the siege of the Legations, and whose interesting account of her work appeared in "The Hospital" Nursing Mirror of January 26th. The King has also accorded the same distinction to Sister Jessie Molyneux Ransome; Miss Lillie Emma Saville, M.D., of the London Mission; and Miss Abbie Goodrich Chapin—the order in each case being given in recognition of services at the International Hospital during the siege. The substantial services of Madame de Ferrières and her colleagues at the French Hospital, Johannesburg, in nursing our sick and wounded soldiers with unremitting devotion have likewise been gracefully recognised by the Sovereign. His Majesty, in bestowing the Royal Red Cross upon the superintendent of a hospital whose nurses were drawn from the staff of the Convent of the Sacred Heart, has shown that he intends to honour those who deserve honour, whatever their nationality may happen to be. There can be no doubt that his action will afford great satisfaction to French nurses in particular, and to the French people in general.

### MORE NURSES FOR SOUTH AFRICA.

We are informed by the Secretary of State for War that the following members of the Army Nursing Service Reserve left England for South Africa in the *Nubia*:—Sisters A. E. Ball, B. M. Bentham, L. M. Biggs, I. Booth, G. S. Edwards, A. E. French, B. Gilbert, M. Glenn, K. F. Grosvenor, N. P. Haine, L. M. Hinton, A. A. Hooker, S. Horsley, H. R. Howard, E. Jasper, F. M. C. Lindsay, E. J. Low, S. A. Marshall, E. Newton, M. Ounsworth, L. Parsons, K. Penman, A. M. Read, I. E. Saunders, S. C. Schliemann, M. Steenson, N. L. Sutcliffe, A. Sutton, and B. F. Whyte. Of these sisters Miss Sara Horsley was trained at Oldham Infirmary, and has since been charge nurse at Hartlepools Hospital, theatre and surgical nurse at Peterborough Infirmary, and head nurse at Devonshire Hospital, Buxton; Miss Esther Newton was trained and has since been staff nurse at the Seamen's Hospital, Greenwich; Miss Bessie F. Whyte was trained at Rotherham Hospital, where she was subsequently staff nurse, afterwards acting as staff nurse at the Royal Chest Hospital, charge nurse at Brook Fever Hospital, and nurse in charge at Hoober Fever Hospital, West Melton, Rotherham; Miss M. Glenn was for some time matron of Mildenhall Cottage Hospital.

### NURSING IN THE UP-COUNTRY TOWNS IN SOUTH AFRICA.

A nursing sister in South Africa thinks that a nurse with some capital might do well in any of the up-country towns where phthisical patients are sent. The climate of Cape Town is not suited to anyone with chest trouble, but an Invalid Home would be a great boon to many persons who are now compelled to live in an ordinary boarding-house. Numbers of these patients land without friends, and sometimes without funds, just in time to die. The latter, when they are too ill to continue their journey are admitted to the hospitals. The places to which consumptive patients are sent are Bloemfontein, where cases are admitted at St. Michael's Home under the sisters; Kimberley, where there is a sanatorium; Beaufort West and Middleburgh, and many other towns where there is no skilled nursing to be had. Those who imagine that South Africa consists almost entirely of rocky mountains, and veldt will be interested in the following account of a garden sent by a nurse, showing how readily the soil responds to cultivation—a fact which should make adventurous spirits welcome the opportunity of settling there. At the entrance there is a kind of moat, with a rustic wooden bridge over it, covered with roses. The banks at the sides are a mass of honeysuckle growing from out of the water, and trees, lovely reeds, and grasses are all in rich profusion. In the garden are citron, orange, lemon, peach, nectarine, and the ordinary fruit trees laden with fruit, and almost every kind of flower which one can think of, with rows upon rows of tuberoses growing in the open as irises grow at home. The garden is only about eight years old, and nearly everything has been planted by the owner. The hills around resemble the South Downs of England, and the scent of the pines is most delicious.

### THE SERVICES OF NURSES TO THE ARMY.

Several civilian nurses have been mentioned in the South African despatches and five Army Nursing sisters, but we are not surprised that the chairman of the governors of the South Devon and East Cornwall Hospital, Mr. Soltau Symons, should have taken occasion at the annual meeting of that institution to give expression to the feeling that, so far, the services of the nurses who had gone to South Africa have not been recognised as they deserve. Mr. Symons mentioned that nine nurses trained at the South Devon and East Cornwall Hospital have been, or are, at the front. Two went direct from their duties at the hospital, and were specially selected to serve on the staff of the Imperial Yeomanry Hospital in Pretoria. One is at Standerton, one at Bloemfontein, one at Newcastle, one at the hospital at Mooi River, and one whose whereabouts is not at present certain. Two have returned home, one being invalided, and the other is at present engaged at the military hospital at Stoke. Naturally, therefore, the governors of the hospital are interested in the matter, and cheered the remarks of the chairman when he said:

> I have read speeches of the generals and many others returned from the seat of war; they spoke in eloquent terms of the bravery and endurance of our troops; but I have looked in vain for one word of praise for the 500 trained nurses who have risked their lives to nurse the sick and wounded, who have endured the greatest hardships, the greatest discomforts, and have shown true patriotism and loyalty. It is to be hoped that after the war the invaluable services these nurses have rendered to the Army will be fully acknowledged by the Government.

To this we may add it is incredible that the Government will fail in the obvious duty they owe to nurses no less than to officers and men.

### A WARNING TO NURSES LEAVING ENGLAND.

THE importance of written agreements has lately been pointed out in "THE HOSPITAL" NURSING MIRROR, and a case painfully illustrating the deplorable consequences which may arise in the absence of them has just been brought under our notice. A nurse left for India with the family of an officer without any written agreement about being sent home. She was not required for the full period of her engagement, and the people with whom she went out were glad that her services should be turned to account by another officer, who is said to have undertaken that the terms made with the first man should be carried out. This, however, was not done, and the unfortunate nurse is now at so great a distance from her home that it will cost her 400 rupees to get there. A little charitable assistance has been given her, but she will have to pay practically the whole of this sum out of her slender savings. We hear that she had a verbal understanding, but her sad experience will, we hope, prevent nurses who read it from accepting any engagement out of England without a written agreement.

### THE NEW LADY SUPERINTENDENT OF MELBOURNE HOSPITAL.

THE appointment of Miss Burleigh as lady superintendent of Melbourne Hospital was announced in our columns some time ago. The *Melbourne Age* of January 21st says:—"The selection was made at a meeting of the hospital committee specially convened for the purpose, but attended by only twelve of twenty-two committeemen. The list of eight candidates was speedily reduced to two ladies—Miss Jane Walsh, of Newcastle, New South Wales, and Miss Burleigh—about the relative qualifications of whom there was an equal division of opinion, the successful candidate being finally chosen on the casting vote of the chairman. After describing her career, the *Age* adds: "Miss Burleigh bears a high reputation as a disciplinarian among nurses, and as a general manageress of all details affecting the welfare of hospital patients."

### NO TRAINED NURSES AT WESTMINSTER WORKHOUSE.

AT an inquest on Wednesday on a private in the Duke of Cambridge's Imperial Yeomanry, who was found lying in Leicester Square in an unconscious state, and was taken to the Westminster Workhouse, where he died, the coroner expressed surprise on finding it given in evidence that there are no trained nurses in the workhouse—a surprise which, we think, will be shared by our readers in view of the kind of cases that are taken to the institution in Poland Street. The untrained nurse for the six male wards has sixty "patients" to look after.

### A TRIBUTE TO THE LATE MISS HANAN.

A FRAMED picture, enlarged from a photo, of the late Miss E. G. Hanan, formerly lady superintendent of the Crumpsall Infirmary, Manchester, was recently forwarded by Miss M. D. Farquharson, lady superintendent of the Melbourne Hospital, and Mrs. T. G. Leslie (formerly Miss M. E. Stephenson, of Cork), matron of the Victorian Eye and Ear Hospital, Melbourne, Australia, to Major Ballantyne, governor of that institution, with a request that it might be hung in the nurses' sitting-room as a token of esteem and affection for their late excellent superintendent, they having had the privilege of being trained under her.

### THE MYSTERY AT NEWTON ABBOT.

THE promised inquiry at Newton Abbot Workhouse Infirmary by the guardians cannot be regarded as satisfactory. As it will be remembered, Miss Flaherty was called upon to resign without any reason being given to the Board, and three other nurses tendered their resignation with her. The inquiry has revealed the fact that the nurse declined to be responsible for the night nursing of an old woman of 90 who, to quote the medical officer, "had lately shown considerable mental excitement, became at times restless, delirious, and noisy, but on account of the feeble state of her health and more than once, the near approach of death, it had become out of the question to send her to an asylum." But her responsibility appears to have been insisted on, notwithstanding that it was admitted that she had to take charge, in addition, of six wards with 56 patients, for whom she was also responsible. The doctor, it has transpired, gave instructions that the old woman was not to be left alone, but that either the nurse or the wardmaid was to remain beside her, and if more help was required to report to the superintendent nurse. At 5 A.M. the patient was asleep, and the nurse, having to discharge duties, for which she required assistance, in another ward, went there and took the wardmaid with her. In their absence the old woman got out of bed. Undoubtedly, the nurse erred in so doing, but would she not equally have erred had she neglected her duties in the other ward? Surely such a breach of discipline does not call for dismissal—which is practically the action the Board have taken. Are there no extenuating circumstances? The nurse, we think, acted straightforwardly when she said she could not attend to this particular patient and do justice to the rest under her charge. The magnitude of her offence is that she left the one in the interest of the rest, but that arose from her being given an impossible task. Since then an attendant has been continually with the old woman to prevent her from falling out of bed. The Board did not trouble themselves to inquire why it is reasonable for a nurse to protest against being saddled under such circumstances with the responsibility of an admittedly weak-minded and troublesome person. We suspect that the root of the mischief lies in the fact that the nursing wards of hospitals are used as lunatic wards, and that nurses do not expect to do the work of lunatic attendants. It is significant that Dr. Ley, who was so prominent in bringing the light of day on scandals at Newton Abbot some years ago, has protested against the Board's action in asking Miss Flaherty to resign. The three other nurses came before the Board, but the chairman would not allow them to state why they had tendered their resignations, which were accepted, Dr. Ley characterising it as a gross injustice. We understand that none of the nurses who resigned has been in the infirmary as long as six months.

### INFIRMARY NURSES AND WOMEN GUARDIANS.

THE subject of women Poor-Law guardians was discussed by members of the Women's Co-operative Guild last week in the Conference Hall of the Co-operative Wholesale Society, Leman Street, E. Among other points raised were (1) the position of an infirmary nurse; and

(2) the training of matrons. Miss Llewelyn Davies (general secretary) said that the character of workhouse inmates was changing every year; workhouses were becoming increasingly hospitals for the sick and infirm. This made women guardians all the more necessary. Although much of the nursing used to be done by old and imbecile paupers, this condition of things was now largely done away with. Most infirmaries now had a staff of trained nurses, and many had a specially skilled children's nurse as well. She urged the need of women inspectors, and spoke of Mrs. Nassau Senior's work in this direction. The post Mrs. Senior held had been allowed to lapse, and it ought to be revived. Usually, the inspector made his rounds in company with the master, and this made it almost impossible for the nursing staff to speak to him on any matter on which advice was needed; but if the inspector were a woman, it would be much easier. The large number of women officials made it obvious that a tactful woman who used her influence wisely would be of inestimable value to the Board. Mrs. Truscott, member of the Board of Guardians for West Ham, said there was a woman inspector at West Ham, and that she did her work very thoroughly. A new infirmary was being built: it would be, as the infirmary always should be, apart from the rest of the building. In this case she thought it was almost ideal, for it would stand close to what had been a gentleman's house in its own grounds.

### A SHAM NURSE SENT TO HARD LABOUR.

The number of sham nurses has at any rate been temporarily reduced by one. A young woman who in 1899 called herself Nurse Macdonald and said she was at Charing Cross Hospital, and later on posed as Nurse Watkins, of Netley Military Hospital, pleaded guilty last week at Westminster Police Court of a number of small frauds, and was sentenced to hard labour for two months. Of course she was never at either Charing Cross Hospital or Netley, and it appears that her real occupation four years ago was that of a general servant. Her history since then has been strangely chequered, but as lately as June last she was living in Montague Mansions, London, and describing herself as Sister Macdonald, and a niece of Major-General Hector Macdonald. This, like most of her other stories, was false, and the only thing that can be vouched for is that her moral character is not without flaw. It seems strange, however, that when she endeavoured to obtain goods as a member of the nursing staff at Charing Cross Hospital she was not brought to book. The simple method of an inquiry at the hospital would have disposed of her pretensions.

### PLYMOUTH WORKHOUSE INFIRMARY.

The medical officer to the Plymouth Guardians has performed a public service by drawing attention to the inadequacy of the accommodation at the Workhouse Infirmary. It may be argued that his half-yearly report tells them nothing that they did not know before, but it is very evident that the Board require waking up to their responsibilities. Three years ago the guardians were advised that new infirmary nurses' quarters and other improvements were a necessity; but they have made no real effort to relieve the congestion. Dr. Cook now points out, among other things, that the accommodation during the past six months for the several classes of sick has not been sufficient: the sick wards were often overfilled; the nursing is satisfactory as far as it goes, but thorough supervision is impossible because of the number of the wards and the fewness of the nurses; the lavatory accommodation is insufficient, bath accommodation is very poor, there is no hot water laid on to the lying-in ward, and there is want of proper ward, kitchen, pantry, and scullery accommodation. These few excerpts will give a general idea of what the whole place is like and how lacking it must be in comfort to patients and nurses alike. Nurses have to cross open yards in all weathers from one part of the hospital to another. What night nursing is under such conditions can easily be imagined—a trying ordeal if not a source of physical discomfort. I is not surprising that in these circumstances changes in the nursing staff are frequent, and the new superintendent nurse, to whose appointment we referred last week, will find that she has her work cut out for her.

### CROYDON INFIRMARY.

No reply has yet been received by the Croydon Guardians from the Local Government Board as to the differences between the guardians and the matron. With the object of bringing the matter to a determination, Mr. A. G. Sibun placed the following motion on the agenda for Tuesday's meeting:—

> That in view of the present strained relations between the matron of the infirmary and the Board, also between her and the chief officers of the infirmary, which seriously interferes with proper administration at the infirmary, Miss Julian be requested to resign her appointment as matron of the infirmary within a period to be named by the Board, and that, failing a compliance with this request, an appeal on the subject be forthwith made to the Local Government Board.

Mr. Vinden said in the absence of Mr. Sibun he would move the resolution. Mr. Stevens seconded. Mr. W. H. J. Owen supported, and said there was friction going on all day long. Mr. Shirley deprecated these resolutions as keeping alive the agitation and friction. It would be better to let everything remain in abeyance until they knew the decision of the Local Government Board. The Board rejected the resolution, five voting for it and seven against. Mr. J. W. Gardiner, earlier in the afternoon, took exception to a statement in "The Hospital" Nursing Mirror, and thought the Board should deal with it. By a majority the Board decided not to interfere.

### SHORT ITEMS.

An interesting account is given in a Natal paper of Christmas festivities arranged for the soldiers' wives and children of the Camp, Fort Napier, got up under the auspices of Miss Brown, the Alexandra nurse, and some of her friends. No less than eighty happy persons of various ages were entertained at a garden party and tea in the marquee of the Soldiers' Institute at Maritzburg.—The Joint Committee of the Women's University Settlement, Southwark, the Charity Organisation Society, and the National Union of Women Workers, have arranged a course of six lectures, to be given by their staff-lecturer, Miss M. McN. Sharpley, on "Earning and Spending," at the Westminster Town Hall on consecutive Thursdays at 11.30 A.M.—The annual general meeting of the Nurses' Co-operation will be held on Tuesday next, at 5 P.M., at 8 New Cavendish Street.—We regret to learn that Sister Lucy Bulkely Williams, of the Army Nursing Service Reserve, is dangerously ill of enteric at Johannesburg. The condition of Sister Ward is reported to be improving.

### Medical Lectures to Nurses.

By CARSTAIRS DOUGLAS, M.D., B.Sc. Edin., F.R.S., F.F.P.S. Glasgow, Professor of Medical Jurisprudence, Anderson's College Medical School, and Dispensary Physician, Samaritan Hospital, Glasgow.

### I.—HÆMORRHAGE FROM THE LUNGS.

So intimately is the blood bound up with our idea of vitality that any sudden and excessive loss of it fills us with apprehension and dismay, and calls for prompt and effective action lest life itself be the penalty. I do not purpose speaking in this place of ordinary primary or secondary hæmorrhage from external wounds, or as a result of operative interference, since this aspect of the subject can be more suitably dealt with in surgical lectures. But not less serious and alarming are hæmorrhages from the lungs, the stomach, and other internal organs, and it is well that a nurse should bear clearly in mind the nature, cause, source, and treatment of such bleeding, for the successful control of it may lie entirely in her hands in the absence of a medical man.

*Hæmorrhage from the Lungs.*—Whenever a patient spits or coughs up blood we use the term "hæmoptysis" to designate the condition. In many cases the bleeding is slight, as in certain ulcerations of the larynx, in early tubercular disease of the lungs, and where there is pressure on a bronchus or bronchiole from a new growth or foreign body. In all these cases blood-stained expectoration may be seen, but the bleeding is seldom of such a degree as to constitute a menace to life. Again, a nurse may frequently note, in cases of mitral disease, where there is regurgitation into the left auricle, and thence through the pulmonary veins to the lungs, that the patient coughs up more or less viscid sputum distinctly tinged with blood. This results from the great engorgement of the lungs, and the hæmorrhage is rather a relief to the patient than otherwise. But there are two conditions in which pulmonary hæmorrhage is profuse and, indeed, terrifying. The first of these, and one which practically invariably proves fatal, is where an aneurysm of the aorta ruptures into one of the bronchi. Here, with awful suddenness, there may be a great gush of blood from the mouth, while at the same time the air-tubes become entirely blocked up, and the patient succumbs within a minute or two from the combined effects of hæmorrhage, asphyxia, and shock.

The second condition (by far the more frequent) in which free bleeding from the lung may occur, is ordinary pulmonary phthisis, or "consumption" in the everyday use of the term. For practical purposes it may be stated that it is only in the later stages of this disease that alarming hæmoptysis is apt to occur. Certainly there is often slight spitting of blood in the earlier months of the illness, but at this time it usually arises from the congested and swollen bronchial mucous membrane, and is seldom great. One may bear in mind two truths regarding hæmoptysis in phthisis: (1) It occurs sometime or other in the great majority of cases, and (2) it tends to recur. In the later stages of the disease, when cavities or "vomicæ" are present, the bleeding is much more serious, and may even prove fatal. It owes its origin either to an erosion of or ulceration into a large vessel lying on the wall of a cavity, or to the rupture of small aneurysmal dilatations which are very apt to form in the course of such vessels. In the latter case the hæmorrhage may follow immediately after some sudden exertion. The patient then becomes aware of a warm salt taste in the mouth, and forthwith expectorates a large amount—it may be a pint—of more or less florid blood mixed with mucus, saliva, or perhaps pus. The loss of such a quantity is a serious matter to an already enfeebled and anæmic patient, and it is not surprising that it should frequently precipitate a fatal result. In other cases the hæmoptysis is less profuse at any one time, but recurs with alarming frequency, impressing upon nurse and friends alike a conviction of the gravity of the situation.

What, then, should a nurse be prepared to do in a case of marked hæmorrhage from the lung, especially where a medical man cannot be procured for some hours? It is obvious that, unlike surgical bleeding, the flow cannot be stopped by any method involving direct pressure on the bleeding point, or ligature of the vessel of supply. Nature herself must form the thrombus, or plug which is to close the aperture in the vessel, and what we have to do is to aid her efforts in every way. The patient should be placed supine on a bed and kept at perfect rest, so that the blood-pressure may be reduced to a minimum. The room should be quite cool and kept darkened. Visitors must be excluded and conversation forbidden. All bodily exertion must be lessened as far as possible, and for the requirements of the bladder and bowels a bed-pan should be used. The diet must be of the lightest and most non-stimulating description, chiefly milk and barley-water, and a feeding-cup should be employed. For the thirst that not unfrequently follows the patient may have small chips of ice to suck. The application of an ice-cap to the chest is advocated by some, but it is doubtful if its action can penetrate as far as the ruptured vessel: it may possibly prove of some service by calming the heart's action, and so lowering blood-pressure.

The styptic drugs commonly employed for internal hæmorrhage are of little use here; indeed, there is but one medicine which is really reliable, and that is opium or one of its preparations. By soothing the patient, preventing restlessness, and subduing cough it may prove of much value. Twenty minims of nepenthe or of laudanum, or ten of Battley's solution or of chlorodyne, may be safely administered, and repeated in four hours if necessary. Ergot should be avoided. Where the hæmorrhage is protracted and tends to recur the bowels should be emptied, first by a glycerine enema or suppository, and in a day or two by a saline purge.

### Minor Appointments.

ROYAL ALBERT EDWARD INFIRMARY, WIGAN.—Miss Dorothy Caffery, Miss Mabel Cotton, Miss Anne Darlington, and Miss Alice Edith Keeble have been appointed sisters. All four were trained for three years in the same institution.

HUNGERFORD UNION.—Miss Mary Fraser has been appointed nurse. She was trained by the Meath Workhouse Nursing Association at the General Infirmary, Worcester, and has been assistant nurse at Newton Abbot Infirmary.

CHESTERFIELD HOSPITAL.—Miss Lilian Shepherd has been appointed sister. She was trained at the Leeds General Infirmary, where she was afterwards charge nurse, and she has since been sister at Colchester Home for the open-air treatment of phthisis.

LONGMORE HOSPITAL, EDINBURGH.—Miss Ira Martin has been appointed staff nurse. She was trained at the Western Infirmary, Glasgow, and has since been assistant matron at Broomhill Home, Kirkintillock, and theatre sister at the Hospital for Women, Shaw Street, Liverpool.

### Parish Nursing in Germany.

By an English Sister.

Perhaps it will interest your readers to know the ordinary duties of a parish nurse in the Mecklenburgische-Marien-Frauen Verrin v. Roten Kreuz (red cross nurses). All the nurses in the Marienhaus, also called Mutter-haus = (mother house), are supplied with coffee, with a roll and butter. Butter is an exception with coffee in the north of Germany, and in most hospitals and nursing institutions neither butter nor sugar is allowed. At 7 a.m., the daily work of the parish nurses begins.

### The Arrangement of Districts.

In Schwerin the town council pays an annual subscription to our committee, which then provides the parish nurses for the town. Schwerin is divided into ten districts which are looked after by men especially elected by members of the council and the chief parishioners in the districts. These districts are again divided into quarters, and certain men are appointed over them by the district Vorsteher, or chief members, the latter being called Armen-pfleger, or "nurses of the poor." The Vorstehern and Armenpflegern are responsible for the well-being of their district; the poor and needy send to the Armen-pfleger, who goes personally to see what is wanted, and then he reports to the Vorsteher, who, if necessary, writes out a form asking the poor or district doctor and the parish nurse to visit the family. Without these forms we are not allowed to visit any poor person. Our work does not consist exclusively in nursing, for often we are sent to people in want only. For these cases we are able to give meat, milk, and bread tickets, and try to find them work to do, &c.; but one has to be careful and to possess plenty of tact, as it is not easy to know really if the people are in want through idleness or through not being able to find work. Therefore one has to know the people well first. Then we often have old people to look after, old women who are not able to work any more, but who are not bad enough to be sent to the workhouse, for the workhouse here is different from ours in England—vagabonds and waifs are the general occupants—so we give these poor old people our help.

### The Daily Rounds.

But to go back to our daily rounds. At 7.20 a.m. we start, and generally go to the most serious cases first, take temperatures, wash the patients and comb their hair, make the beds, and if there are no relations or kind neighbours to look after them a little, we wash the floor—carpets and oilcloth are as yet unknown luxuries here among the poor, so one is provided with a scrubbing-brush attached to a broomhandle, and a thick cloth for swabbing the boards—tidy up the room, &c., and go on to our next case. Very often we have an old woman to care for, so after getting her ready and tidying the room we boil her dinner, wash up, &c., and try to make her comfortable for the rest of the day; usually some friend or neighbour looks in afterwards and puts her to bed and gives her supper; if not we must. Then, perhaps, we find a large family of small children, the mother is ill or very delicate, and the father at work all day; here there is plenty to do—wash the children, either wash the babies or see that the mothers do it properly, make the beds, and do the heaviest work; or we find a family where the chief members are ill or recovering from severe illness, and who chiefly want strengthening things; they, too, must have meat tickets, or wine, eggs, soup, &c. Everywhere I notice we have to open the windows; one would think in a country where no open fireplaces are used, and where there are no ventilators, the people would, of their own accord, let in a little fresh air, but it is a very rare occurrence if they do, and one can be almost certain that as soon as Schwester has left the house, sun and air are promptly shut out again.

### Our Meals.

Then at 1 p.m. we have dinner—soup, meat, and vegetables. At 4 p.m. coffee and rolls; directly after we start out again, settling our patients for the evening or night, visiting those who only need to be visited two or three times a week, give out the Sunday papers provided by the town for that purpose, settle accounts with the tradespeople who have orders to give goods to the ticket-holders, &c. In fact, after dinner and coffee, we do all the extra work, as we call it, for naturally we are very busy all the morning. Then supper at 8—tea, bread and butter with German sausage, cheese, &c.—bed at 10.

### Paying Patients.

However, sometimes we hear of a patient who only wants a nurse for an hour or two daily, for there are some things which servants, no matter how willing, cannot do, so if we can manage it we go; perhaps it is to assist at an operation performed in the house, or perhaps to make a bed for an invalid whom it is difficult to move. Then we charge so much according to the patient's means, never more than 2s. an hour, though persons may give more if they choose. We also undertake night duty, if we feel we can do our duty as well in the day time. I am sorry to say I find I can only do day work or night duty, though I often used to do both; we all like that, as night work is 3s. per night, and the extra money a parish nurse earns she is allowed to keep for her parish and not give to the Marienhaus, provided, of course, it is spent on the parish only. On the whole, there is no actual poverty in Mecklenburg as in London. The town is small, of course, but besides there are so many people who are really interested in the poor and give money and time. Still, there is no place like England. One sees that more and more as one learns to know the customs and ways of people in other countries.

### Death in our Ranks.

We much regret to record the death of Miss Mabel Cook, matron of the British Lying-in Hospital, which occurred on Saturday night at St. Mary's Hospital. Miss Cook was taken to St. Mary's on Wednesday evening last week suffering from erysipelas and pleurisy, and her illness was thus really of only three days' duration. She was appointed matron of the Lying-in Hospital in March, 1898. Since then the work has rapidly increased in quantity and efficiency, until now it has almost doubled. Miss Cook was trained at the Soho Hospital for Women and at St. Mary's Hospital, where she was sister of the Obstetric Ward for some three years, and gained the highest esteem of the medical and nursing staffs. She held the certificate of the L.O.S. Not only at the British Lying-in Hospital, but also in the nursing world generally, she was regarded with much respect, and there is no doubt that the admirable institution with which she was associated will suffer severely by her death. The Board of Management under whom she worked with so much vigour and self-sacrifice thoroughly recognised the value of her qualities. Prior to the burial at Abney Park Cemetery on Thursday there was a memorial service in the chapel of St. Mary's Hospital on Wednesday afternoon, at which many members of the nursing profession, as well as of the staff of St. Mary's and the British Lying-in Hospital, were present.

We also regret to learn that Miss Edith Wooll, of Guy's Trained Nurses' Institution, died on Tuesday, February 5th, after a week's illness of acute pneumonia.

### A Practical Memorial.

A beautiful wreath was sent by the women of Newry to Queen Victoria's funeral. The wreath and expenses amounted to £7 5s. 3d., and there was a surplus of £7 15s. on hand, which it was decided by the ladies who received the subscriptions to give to the work of the Queen's Jubilee Nurse in the town. Thus, the Queen's memory was honoured in a two-fold manner. The nurse fund has derived further substantial benefit from the offertory at St. Mary's parish church, which was devoted to it on the occasion of the memorial service.

### Nurses and the Industrial Laws.

THE subject of Mrs. Tennant's lecture to members of the Royal British Nurses' Association at 10 Orchard Street on Thursday was "Nurses and Industrial Laws."

Mrs. Tennant, who was introduced by Miss de Pledge (now Mrs. Latter), said she hoped that those present would help to form a link between the factory inspectors and the factory workers. Girls and women in factories and workshops were at the mercy of their employers; and though there were many exceptions, the conditions under which, as a rule, they worked, made it very difficult to obtain for them the benefits of the law. Such a link was necessary, because (1) the number of inspectors was very inadequate, and (2) they had to meet certain difficulties arising out of the fear and ignorance of the workers. "I will keep no one who tells the truth to the Inspector," were the words with which a girl had been dismissed, after giving information about illegal overtime; and it was because of cases like this that they were afraid of making their grievances known. Such ignorance was not unnatural. They learnt nothing at school of the laws under which they were going to work, and the employers' information when engaging them was frequently misleading. There was a stringent rule that a copy of the Factory and Workshops Act should be hung in a conspicuous place; but its language was legal, and it was often hung where it could only with difficulty be read; sometimes it was put away in a drawer. London workgirls must not be compared with the textile workers of the north, whose superior conditions make them well able to protect themselves by combination.

### How can Nurses Help?

The special mission and opportunities of nurses, the lecturer continued, fitted them to help in the formation of the necessary link. They must frequently, in the hospital or the district, come into touch with abuses, though they might not always know such abuses to be illegal. Certain factories were known by certain hospitals as slaughter-houses, on account of the number of accidents that happened there from machinery, &c. Again, a district nurse might be attending a case at home, where she came to know the conditions under which the patient, or some of the family, worked and lived; and if any case of breach of the law came within the nurse's knowledge, she need have no fear that she would be unduly interfering by making it known. There were rights which every member of the community ought to enjoy. There was an enormous gap between the state in which the workers ought to live and that in which they really did live; and by being willing to act as correspondents for the Industrial Law Committee, nurses could help to throw a bridge across this gap.

### Points to Remember.

After explaining certain technical points, *e.g.* the distinction between a factory and a workshop, and between a child, young person, and woman, Mrs. Tennant spoke of the law regarding cubic space as one that especially concerned nurses; 250 cubic feet were required for each worker in the daytime, and 400 if overtime were being worked with artificial light; ventilation, cleanliness, provision against fumes and dust, and sufficient, suitable, and separate sanitary accommodation for both sexes, were all provided for in the Act, and the improvement in the moral tone of a factory, when these conditions were insisted on, was remarkable. The proper fencing of machinery and notification of accidents were matters more commonly neglected than almost any other; a nurse who helped to get an accident reported so that the case was taken up by the Home Office, would be benefiting, if not the worker injured, at any rate her relations and fellow-workers. The temperature of workrooms, hours and meals, and the complicated law with regard to laundries were then touched upon, and—a matter that specially interested nurses—the law that women should not return to work for four weeks after childbirth; this was often broken by the women themselves.

A nurse, said Mrs. Tennant, working where any dangerous trades were carried on, should find out what the regulations were regarding them; they applied, generally speaking, to cleanliness, baths, the washing of hands and cleaning of teeth (in such trades as lucifer match-making, &c.), the taking of meals apart from the workrooms, and the extraction of fumes and dust. The list contained about fourteen such trades. The abuse of the Truck Acts was more common in the country than in London; any case of illegal fining should be reported.

### How to Give Information.

Unless the case were one of very great urgency, such as illegal overtime to be worked that night, it would be well to send the information (which should be very precise as to time, place, and the exact floor of the factory where the law was being broken) direct to the Industrial Law Committee. If very urgent, the Inspector's address should be obtained, and a telegram sent. The advantage of sending to the Committee was that Mrs. Tennant and Miss de Chaumont (the one ex- H.M. Superintending Inspector of Factories, and the other Inspector of Public Health) were on the spot, and might be able, from long experience, to save the inspector much time and trouble, and to simplify her work. When possible it was desirable that the name of a worker ready to give information, if asked, should be in the possession of the Committee, so that the inspector might know with whom it was worth while to talk. If the point was one regarding meal-hours, which were distinctly laid down by law, it was necessary to know whether the time was to be curtailed at the beginning or the end of the period; all such details, though they might sound unnecessary, were what made it possible for the inspector to do her work. Besides undertaking to be correspondents, the Committee would be very glad if nurses whose work gave them these opportunities would help in organising meetings in their district, and in the distribution of literature; copies of all publications of the Committee would be gladly sent them.

### An Indemnity Fund.

There was one very important provision which might relieve the anxiety of any about to undertake the work of correspondents—viz. that an indemnity fund had been started for the workers, in order that any girl or woman giving information with regard to breaches of the law should suffer no loss. There was great need of such help as nurses could give in the way sketched, and by giving it they would benefit not only the individual workers known to them, but also the mass and the future welfare of the race.

A hearty vote of thanks was proposed by a lady, who said that the information given by Mrs. Tennant could not fail to be useful to the nurses present. "Our very work," she continued, "throws us among the poor. District nurses and matrons have special opportunities of noticing breaches of the law, especially with regard to the going back to work of women recently confined. Much tact would be needed, but nurses had special facilities for notifying such cases without involving an one person."

### A Record of Volunteer Nursing.

### CHAT WITH SISTER PATERSON.

By a Correspondent.

Three nurses have just come home from South Africa on board the s.s. *Saint Andrew*, which called at Durban on its way from China. These are Sisters Paterson, Pilcher, and Halkett. With the first I had a few minutes' talk on Tuesday, the day after her arrival at Southampton. This is the second time she has been over with sick and wounded, but the first time she was too unwell for interviews.

### Fever still High.

"Is the fever still very bad in South Africa ? " I inquired.

"The country reeks of it," she replied. "This is the fever season; it is hot in Natal, though so cold in Cape Colony that while there I had a slight attack of bronchitis, which, however, only lasted a very short time. The men are sleeping anywhere, as it is impossible to carry tents with flying columns, and this makes the state of things worse."

"Did you bring home many enteric cases ?"

"Two men developed enteric on board. Otherwise we had not a great deal of illness. A man fell down a shaft and broke his back; he had had two allowances of rum—one more than he should have had—and was on his way to try and get a third when the accident happened. His back was broken, and he is now at Netley. He was a time-expired man, and had no wife or family."

### How they Heard the News.

"When did you leave Durban ?"

"On January the 16th. It has been a very slow passage, thirty-two days; but the boat was dirty, on the outside I mean, and this impeded our progress. Sometimes we were not steaming more than five or six knots an hour."

"Did you hear of Queen Victoria's death at sea ?"

"At St. Vincent we got papers, and these happened to have the King's Army Orders in them. They were read out to the men on Sunday at church parade, and one could see many of them had tears in their eyes. The news had already been signalled to us by a passing vessel, but we did not want to believe it. When we got the papers and saw that it was so, everyone was very much distressed."

"You had left before the news of her illness reached South Africa ?"

"No; we were at Cape Town on Tuesday 22nd, and there was then a rumour of her death; we went to Government House to enquire, and there was no official confirmation, so we hoped it was untrue. She died that evening, and it was a week after the funeral when we touched at St. Vincent."

### Varied Experience of South Africa.

"May I ask how long you have been in South Africa ?"

"Fifteen years. I went out as soon as I had finished my training at Glasgow Royal Infirmary. I was matron of Durban Hospital for some time, and gave up the appointment in order to take up private nursing in Johannesburg. I have been sorry since that I did so."

"Private nursing is rather rough work ?" I suggested.

"It is if you go to the mines; but of course you can choose both your patients and the doctor you work under. There are many very nice people in Johannesburg. Once I went to nurse a doctor who had typhoid in the mines; there were thirty-three people down with it, and I didn't lose one of them."

"That was certainly a record. You must know the country well ?"

"Yes, and the people. Living among them as I did I was aware of the condition of things long before war was declared; as early as June, 1899, I went down to Natal for a holiday, and at Maritzburg offered myself as a volunteer to the P. M. O., though it was not until September 29th that I left Johannesburg to take up army nursing."

### Some of the Earliest Volunteers.

"You must have been one of the first volunteers ? "

"I and Miss Borlase and another friend at Standerton were the first to offer, though of course others have seen as much as we have of active service. Mrs. Ludlow, for example, who was in Ladysmith, as I was, throughout the siege, and is now on the reserve staff; and others as well."

"Are you returning to South Africa ? "

"Yes, but I do not yet know when, nor by what vessel I shall go. It will probably be soon, however."

### Appointments.

Barrington's Hospital, Limerick.—Miss Atthill has been appointed matron. She was previously matron of the Dover Hospital.

Gilmerton Convalescent Home for Children.—Miss K. A. Little has been appointed matron. She was trained at the Western Infirmary, Glasgow, and for the last eighteen months has been staff nurse at Longmore Hospital for Incurables, Edinburgh.

City Isolation Hospital, Wakefield.—Miss Madeleine K. Phillips has been appointed matron. She was trained at Sir Patrick Dun's Hospital, Dublin, and has since been superintendent of nurses, Jervis Street Hospital, Dublin, assistant matron at the Borough Hospital, Wolverhampton, and matron of the Wallsend Hospital, Newcastle-on-Tyne.

Western Fever Hospital, Seagrave Road, Fulham. Miss Laura Clara Clifton has been appointed assistant matron. She was trained at Westminster Hospital and has since been matron at Alcester Fever Hospital, superintendent of the Swansea Nursing Institute, and home sister at Westminster Home.

Isolation Hospital, East Malling. — Miss Margaret Cooke has been appointed nurse superintendent. She was trained at St. Thomas's Hospital, and was on St. Thomas's staff for eleven years. She was subsequently charge nurse at the South-Western Fever Hospital for four years, and home sister at the East London Hospital for Children for five years.

Monsall Fever Hospital, Manchester.—Miss Littlewood has been appointed lady superintendent. She was trained at St. Thomas's Hospital, where she became staff nurse and took sister's duty for the night sister in the Infectious Block and Out-patients' Department and had charge of the Nightingale Home. She was subsequently night sister at the Radcliffe Infirmary, Oxford, sister-housekeeper at Charing Cross Hospital, and assistant matron at the Eastern Hospital, Homerton.

Rose Hill Hospital for Sick and Incurable Children, Babbacombe, Torquay.—Miss Isabel I. Forster has been appointed matron. She was trained at Dover General Hospital, and has since held the following appointments:—House matron and nurse at the West Riding Home for Discharged Female Prisoners, Wakefield; staff nurse at Royal Orthopædic and Spinal Hospital, Birmingham ; charge nurse at Royal National Hospital for Consumption, Ventnor, I.W. ; and during the last four years, matron of St. Mary's Cottage Hospital, Tenbury, Worcestershire.

### TRAVEL NOTES AND QUERIES.

Books to Study (Cairo).—For real useful practical information no book is so good as Baedeker's "Egypt." In the opening chapters they impart a fund of interesting knowledge, but it is an expensive work—15s. I wrote three articles in 1899, which, I think, you might find useful: they were published in the Nursing Mirror of April 1st, 8th, and 15th. If you subscribe to a library, the following are all interesting: "Wintering in Egypt," by Bentley: this is fairly cheap, only 3s. 6d. Cook's "Handbook for Egypt," 6s. A very pretty volume called "Egypt: Descriptive, Historical, and Picturesque," by Ebers. "In Cairo," by Fullerton, 3s. 6d. "The City of the Caliphs," by Reynolds-Ball, 10s. 6d.—this last very good.

### Weddings in the Nursing World.

### MISS DE PLEDGE.

On Monday Miss Josephine de Pledge, late matron of Chelsea Infirmary, was married to Mr. Charles Latter at St. Luke's, Chelsea. It was a very quiet and simple ceremony, only the immediate relatives of the bride and bridegroom being present, but the church was full, nevertheless, for "Matron" has endeared herself to many during her reign at Chelsea, and patients, old and new, and patients' friends were there in force, not to mention every man or woman connected with the infirmary who could by any means be spared, while there were members of the Board of Guardians, and nurses, past and present, not a few.

Members of the nursing staff, in their freshest and whitest of caps and aprons, took the place of a choir, and filled the chancel in a very effective fashion. The whispered comment, "Doesn't Matron look nice!" was well justified, for the bride looked charming as she came up the aisle on the arm of her brother, wearing her travelling dress of dark blue crèpe-de-chine with a toque to match, with touches of old lace in it, and a handsome old lace fichu over her shoulders. The choir sang a favourite hymn, "Peace, perfect peace," the organ pealed wedding music, and the bells chimed at the conclusion of the service, while the nurses ranged themselves along the aisle and made a lane for bride and bridegroom on their return down the church. A reception was afterwards held at the house of the bride's sister, and the nurses had one to themselves in their pretty sitting-room. In the afternoon the bride received the following telegram:—"Long life, health, and happiness to dear matron and Mr. Latter, from her nurses."

Miss de Pledge received a great many presents, including some beautiful jewellery and plate, none of which will be more valued than the gifts which came from those who worked under her at Chelsea. The nurses' present was a silver toilet set; the house and laundry maids gave silver-backed clothes-brushes; the kitchen staff, silver scent-bottle and jar; the laundry women, ivory and silver paper-knife, each sent with a congratulatory address. From "her friends on the Chelsea Board of Guardians" came a silver kettle and stand, and a massive silver bowl from the Executive Committee of the Royal British Nurses' Association, with this inscription:—"Presented to Miss Josephine de Pledge on her marriage by some members of the Executive Committee of the Royal British Nurses' Association as a token of their esteem and appreciation of her good work in advancing the best interests of the Association." Signed by Harriet Costar, Grace Gordon, Godiva M. Thorold, Henrietta Wedgwood, A. E. Fardon, Clement Godson, John Langton, W. Bezley Thorne, and A. T. Schofield.

### MISS HELEN THOMSON.

On Monday also the marriage between Miss Helen Thomson and Mr. Henry Temple Mursell, M.B., was celebrated at St. Stephen's Church, South Kensington. The bridegroom is a well-known surgeon in Harley Street, and the bride, who has lately returned from South Africa, has had a varied experience as a member of the nursing profession. After working in the Children's Hospital, Birkenhead; the Hope Hospital, Manchester; and the Royal Hospital, Portsmouth, she became a private nurse. She has also held the posts of Public Lecturer on Nursing, Secretary to the Royal British Nurses' Association, and Secretary of the Victoria Nurses' Club, London. Miss Thomson, who was given away by Lord Balfour of Burleigh, was beautifully dressed in ivory satin, with a transparent yoke and sleeves of tucked chiffon. Her tulle veil was arranged with orange blossom, and she was followed by five bridesmaids, one of whom was her sister, and another a sister of the bridegroom. They were dressed in biscuit-coloured gowns, the bodices being swathed and trimmed with some exquisite embroidery of the same colour. Round the waist was a deep belt of nasturtium-coloured panne. Although the bridesmaids were attired in colours, the fact of the general mourning had not been ignored, for each of them wore a black hat as well as a rosette of black chiffon on the left of the bodice, from which hung two ends well below the waist. The service was fully choral, and the ceremony was attended by numerous guests. None of the ladies, however, wore nursing uniform.

### The Nurses' Bookshelf.

The Nursing Profession: How and Where to Train. Edited by Sir Henry Burdett, K.C.B. Third Year. (London: The Scientific Press, Limited, 28 and 29 Southampton Street, Strand, W.C. Price 2s.)

The demand for a book like "The Nursing Profession: How and Where to Train," is attested, in a remarkable manner, by the numerous inquiries which are printed almost every week in our own columns from readers who are anxious to enter the nursing profession, and to learn something about particular training schools. We are glad to give the best information in our power on any point; but to those who wish to be always armed with authoritative and exhaustive details, we cannot do better than recommend the acquisition of this unique work. It is literally what it professes to be in the title-page, "a guide to training for the profession of a nurse, with particulars of nurse training schools in the United Kingdom and abroad, and an outline of the principal laws affecting nurses," and though originally it appeared to more than fulfil the purposes of the editor, we notice that concurrently with the third year of publication, there are several important additions. Perhaps no portion of its varied contents is likely to be more appreciated than the introductory chapter on "The Common Requirements of the Training Schools," which, as suggested in the preface, supplies a key to the whole of the volume, "so that anyone who consults its pages may be able to obtain a preliminary knowledge of every requisite particular as to qualification, preliminary requirements, hours of duty, examinations, recreation, salary, premium, sleeping accommodation, pension arrangements, and, in fact, everything which an intending probationer may want to know." The abstract of the principal laws affecting nurses is as serviceable to members of the profession generally as to probationers, and the particulars of societies and institutions, the objects of which include the promotion of thrift among nurses, the raising of their status, the institution of examinations, and the issue of diplomas in subjects which will prove useful to them in their career, cannot fail to prove most valuable. There is a comprehensive index, and the arrangement of the book is as admirably contrived as its subject matter is illuminating and complete.

### To Nurses.

**We invite contributions from any of our readers, and shall be glad to pay for "Notes on News from the Nursing World," or for articles describing nursing experiences, or dealing with any nursing question from an original point of view. The minimum payment for contributions is 5s., but we welcome interesting contributions of a column, or a page, in length. It may be added that notices of entertainments, presentations, and deaths are not paid for, but, of course, we are always glad to receive them. All rejected manuscripts are returned in due course, and all payments for manuscripts used are made as early as possible at the beginning of each quarter.**

### Echoes from the Outside World.

AN OPEN LETTER TO A HOSPITAL NURSE.

ALTHOUGH those of us who witnessed the procession of their Majesties to the Houses of Parliament last week were glad to have done so because of the historical interest attached to the opening of the first Parliament of Edward VII., we were conscious all the time that ours was such a very small glimpse of the gorgeousness attendant on the ceremony. Excepting the coach, everything was necessarily confined to the House of Lords, and many were the wishes expressed on every side, "How I should like to have been in the House!" Still, it was pleasant to note that the King was bright and smiling once more, for the intense sadness of his face as I saw it when he rode as chief mourner on February 4th had been difficult to forget; and the Queen, of course, we had not seen at all since her accession to the title. But on Thursday they were both as public as if they had been in an open vehicle. Almost the whole of the upper part of the wondrous erection was, like Cinderella's coach, of glass. The King and Queen seemed very pleased that their welcome was so hearty, and, indeed, it augured well for their popularity that such a large number of people should have been willing to stand for some hours in the bitterly cold wind for the satisfaction of showing their loyalty.

INSIDE the House of Lords the scene must have been splendid. Of course I was not there, though I have still a vivid recollection of the only time I listened to a debate in the Upper House. I sat in a queer little box, reserved for visitors, on the left of the door, which much resembled the old-fashioned square pews still remaining in a few churches. So I tried to picture what the hall would look like when every nook and cranny was filled with the learning and the flower and the beauty of England. It must have seemed strange to note how the tables had been turned, and that, whereas in most mixed gatherings it is the ladies who supply the colour, on this occasion it was the men in uniform, diplomatic dress or robes, who were "gleaming with purple and gold," whilst the women made a thick black line only lightened by gleaming shoulders and dazzling diamonds. Several little details of the ceremony were worth remarking. For instance, the King, not being entitled till after his coronation to wear the crown, had it borne alongside him by the Duke of Devonshire on a cushion suspended by a support round his Grace's neck. Upon another cushion the Marquis of Winchester—the premier marquis by hereditary right—carried the Cap of Maintenance. These emblems of royal rank may not be touched by any hands but those of the Sovereign. The purple-crimson velvet mantle of the King was four yards long and very ample, lined with ermine and a cape of the same fur behind; the Queen, being a consort, had a much smaller mantle and a much shorter train. Both trains were upheld by young pages of honour in scarlet. The golden chair for the King was, by his express wish, copied exactly for the Queen, that she might as much as possible share his regality. The Royal pair entered the hall hand in hand. When they came to the Throne they bowed to it together, still hand in hand, ascended the daïs, and then the Queen stooped and kissed the hand of her Royal spouse before they parted, and the King stood whilst she took her seat. The reason why the Sword of State was not carried by the Prime Minister was because Lord Salisbury asked to be allowed to be excused from so fatiguing a task. The Sword is a long heavy weapon, sheathed in a scabbard of crimson velvet encircled with gold metal plates, and it has to be held well aloft, so no wonder the Premier deputed his duty to the Marquis of Londonderry.

WEDDINGS have been celebrated in many places the last week or so, and there has been quite a competition to secure a day for the ceremony. The Duke of Westminster's marriage with Miss Sheila West came off on Saturday, after being postponed from the previous Thursday, owing to that date being reserved for the opening of Parliament. The bride looked very beautiful in Empire dress, and all her bridesmaids, even the two little children, were attired in the same style, making in all a charming picture. Instead of bouquets they carried tall branches of Annunciation lilies, set into silver holders shaped like lily leaves, and the bride's train was carried by two small pages, each attired exactly like Gainsborough "Blue Boy." Contrary to the usual custom, the bridegroom took his wife at once to his country home instead of going for the ordinary honeymoon. The tenants met them at the station, and escorted them to Eaton Hall by torchlight. On Tuesday great interest was displayed in the nuptials of Major-General Pole Carew and Lady Beatrice Butler, at the Guards' Chapel. The Chapel is so small that even all the personal friends could not be admitted, but an enormous crowd assembled outside and cheered the bridegroom loudly. You will remember that he was in command of the Guards Brigade in South Africa. Lady Beatrice is a descendant of Anne Boleyn and Queen Elizabeth. A marriage under less happy auspices was that of the eldest daughter of the Queen of Spain, the Princess of the Asturias, with Prince Carlo de Bourbon, which was celebrated at Madrid last week. The ceremony, according to some of the papers, "passed off without incident, and there was no public disorder to disturb it." But this was only done by guarding every approach to the palace with cavalry, and by proclaiming a state of siege in Madrid. Ever since the engagement was made public there has been a ferment in the principal Spanish towns, so little pleasing to the people was the prospect of the alliance. But although unrest is over all the land, it is hoped by the authorities that a revolution will be avoided by paying and pampering the troops in Madrid, for all Spain looks to its capital for its cue, and if nothing serious happens there, the other towns will probably remain quiet.

THE women of Europe will owe a debt of gratitude to the French ladies, Madame Petit and Mademoiselle Chauvin, who have acted as pioneers in the matter of female barristers A little success has more influence than reams of argument and the fact that France has seen fit to admit two lady lawyers, and that their services have been at once in requisition, has evidently had a direct bearing upon the powers in Belgium and Scotland. In the former country matters are as yet in embryo, for the bill to permit women to practise in the law courts is only now before the Belgian Chamber, but as the movement in favour of the innovation is very marked it is thought probable that the measure will meet with considerable support, even if it fail to become law at once. In Scotland Miss Margaret Hall's petition to be allowed to practise has been favourably reported upon by the Scotch Incorporated Society of Law Agents—who are evidently of a more enlightened turn of mind than their brothers in England—so that it is not at all improbable lady lawyers may become, before long, familiar personages in the "Land o' Cakes." At present Miss Hall is awaiting the decision of the whole Court of Session as to whether she may enter for the two preliminary law examinations. But it is strange to learn that Russia, which is generally looked upon as somewhat behindhand in matters of civilisation, boasted of a lady advocate as long ago as 1871, and that she pleaded in the Palata at Nijni Novgorod, with her male confrères thirty years ago.

### Everybody's Opinion.

[Correspondence on all subjects is invited, but we cannot in any way be responsible for the opinions expressed by our correspondents. No communication can be entertained if the name and address of the correspondent is not given, as a guarantee of good faith but not necessarily for publication, or unless one side of the paper only is written on.]

### OVERWORKED ARMY SISTERS IN SOUTH AFRICA

Miss M. F. Lightfoot, writing from No. 6 General Hospital, Johannesburg, South Africa, under date of January 23rd, 1901, says: I have just opened The Hospital of December 29th, and read, to my extreme astonishment, that I have been "very ill." As my only illness during my year in South Africa has been tonsillitis, from which I was off duty in August for exactly one week, I cannot see on what possible grounds the statement has been made. No doubt all the sisters who have been out here for any length of time are beginning to feel the strain of continual work; but I certainly, for one, by no means feel myself "overworked," though I quite agree that if the war goes on for very much longer it would be wiser for some fresh sisters to be sent up to take the place of some who are beginning to flag. We understand that Miss Lightfoot is now on her way home.

### A WARNING.

The Matron of Corbett Hospital, Stourbridge, writes: The following little incident may prevent some hospitals from being imposed upon. One evening last week, about 8.30, a young man came here saying that he had swallowed some nitric acid by mistake for whisky, taking a small flat bottle from his pocket, which was unlabelled, containing a small quantity of strong acid. At first sight he appeared in great pain, and said that it was with difficulty he spoke and opened his eyes. His lips were white with the burn of acid. One of my nurses recognised the man as a patient in a hospital in the East of England where she was some twelve months ago. The man had the same tale to tell, and also arrived about the time of closing the wards. On that occasion he was taken in and treated, as his lips and mouth were marked with acid. He then appeared well acquainted with hospital routine. After having his mouth examined by the doctor here he was quickly sent about his business. His description is: Age about 24, small stature, fair moustache, hair very thin on the top of his head, very clean-looking, and tidily dressed in a brown suit. He evidently wanted to get comfortable quarters for a few days.

### NURSES' UNIFORMS.

"M. A. H." writes: I am sorry to see by "M. E. R.'s" letter that the uniform is so abused. At one time it was used in Manchester to such an extent that I gave it up, and would not be seen in the street with it on. Some years ago a lady in London sent a letter to The Hospital saying she preferred to see her maids going out with uniform—it made her house look so respectable. Why cannot we have a uniform kept, and only allowed to be worn when on duty by nurses, and only sold to them by Government—the same as the soldier and the policeman? I for one am ashamed to be seen in uniform when I see the so-called children's nurses with it on in London, where the practice obtains more than in Manchester and Liverpool. I trust someone will take up the question. There is a body of nurses strong enough to fight it out.

### SISTERS AND PROBATIONERS.

"Veritate Vincimus" writes: Reading some letters under this heading, I venture to put forward some ideas which may not have occurred to "Senior." By the *nom de plume* she adopts I infer that she is no "novice" in the nursing world, and may, during some part of her career, have undertaken a "sister's" duties, presumably, temporarily. If she has done so, she will understand the enormous responsibilities attaching to that post. For all that takes place within that little sphere of hers a sister alone is accountable to her matron, medical men—in fact, to everybody who is in higher position in hospital than herself. If she has not loyal and capable nurses under her (the more refined the better), the work, which is of necessity more or less heavy, assumes gigantic proportions. I think, should "Senior" reflect, she will realise that her statement of what one "sister," and presumably her own "sister," says, is not a guarantee that all "sisters" are alike; nor should the one she refers to, who has erred—shall I say from a mistaken sense that she was furthering our noble cause?—by removing her so-called "learned ladies" from the thorny path of the nursing work, have been referred to. Probationers are but human, and of all degrees of smartness; but those one appreciates and likes to have work for one are the neat, quiet, quick, and always (when on duty) at work, of retentive memory, methodical, sympathetic but firm with the patients, up to time in their work, and ready and cheerful when "sister" wants them, and who care to watch and observe what is required of them in the wards. Those probationers who are non-observant, slow, and unrefined in manner cause friction in the wards they may be placed in, which is only removed when they improve. Their mistakes are of necessity numerous; but if they give themselves up to their work, and heartily and thoroughly care for all and every detail that is told them, at the end of two months they should make careful, orderly, and refined nurses in embryo. It is a pity, to my mind, that "Senior" laid herself open to the suggestion of want of, on her part, "hospital" etiquette.

### VISITORS FOR THE UNVISITED.

"J. H." writes: I have read with interest in your paper about a movement called "Visitors for the Unvisited." This is a new and beautiful workhouse infirmary in Lancashire: it holds about 220 beds, divided, roughly speaking, into four fine wards, with their accompanying isolation wards. The building has been open a little more than five years, and for the last four years we have had a band of young lady visitors organised by the lady guardians to visit in the infirmary. The first year was only a trial, but now at the end of the fourth the thing is established and a great success. The lady guardians began in this way: they asked a number of young girls of nice position from all the districts in the union to visit at the infirmary. The visitors were to take a month in the year and to come up on a set day for about two hours at a time, cards being provided to this effect and given to the visitors. A visitor could bring a friend, or send a substitute, or change her day by communicating with the head nurse, and about four visitors were arranged for in each month, so that no ward should be missed out. The lady guardians agreed to meet the lady visitors annually at the infirmary to arrange for the ensuing year, vacancies having arisen and new members being brought up for proposal. Preference was given by the lady guardians to young girls, in order that their fresh young faces might help to cheer the sick and bring something bright into their lives; and because (and with all due deference to the older or married people) they would not take upon themselves to interfere in any way; and I, the superintendent nurse, may truly say, at the end of the fourth year, that I have never had a word of complaint or any difference either with the patients, nurses, or doctors with regard to the young lady visitors. It is true that just at the first I made it my duty to take the visitors in the wards to give them little hints, but more in case of their feeling shy or getting a shock when brought face to face with real illness; and I may add that very few have given up because they disliked visiting. The lady visitors may only bring books, flowers, a few sweets, or fruit, and they are not expected to go into the isolation or maternity wards. This is clearly understood. At Christmas the visitors have always helped most kindly with the "tree" in the children's ward, many giving nice gifts for it, others volunteering to assist to decorate; and from time to time in the winter months a few have banded together and come up and given entertainments to the patients: these are much appreciated. I need hardly say that the patients, specially those who are in a length of time, know all the visitors and look eagerly for them, and are disappointed if by any chance they do not come. No one could call our wards "dreary," nor take any exception to the nurses: they are almost invariably sensible, kind, nurse-like women, doing their work with a quiet dignity which puts them beyond dispute. Personally I think young lady visitors are an acquisition to an infirmary, and I hope other institutions will try the same scheme when they read how successful this has been.

### A Book and its Story.

### THE POWER THAT IS WITHIN.

THE WORLD BEAUTIFUL. By LILIAN WHITING. (London: Gay and Bird.) Price 3s. 6d.

"THE WORLD BEAUTIFUL" is a delightful book. We have chosen it for notice this week, not because it is a new book, as the edition before us bears the imprint of the ninth, but as being one that we hope may be especially helpful to readers of THE HOSPITAL, either for their own perusal or for the use of convalescent invalids in private nursing. It will be obvious to anyone reading it first why this distinction is made; it is written for educated, cultivated readers. But there are many passages that would be suitable for reading to sick folks of any class, although it does not deal with sickness, but is addressed primarily to people living in the world—to ourselves, who go in and out, who pass without touching those we meet. "The world beautiful," as the authoress believes, and as we know, is not without us, but within. That each one should keep the light that is within them clear and bright, so that its rays may warm and cheer all with whom they come in contact, is the object of her helpful little book. It has no story to tell, but it has a message, a bright and cheery one, whose tone is sound, and whose counsel is tempered by "saving common sense."

"The Duty of Happiness" contains many sensible reflections; for instance, "Happiness, like health, is the normal state: and when this is not felt, the cause should be looked for, just as in illness the causes should be scrutinised and removed.

"Like the Kingdom of Heaven the World Beautiful is within; and it is not only a privilege but an absolute duty so to live that we are always in its atmosphere. . . . After all, it rests with ourselves as to whether we shall live in a World Beautiful . . . in the sweet, sunny atmosphere of serenity and light and exaltation, in that love and loveliness that creates the World Beautiful."

"Let no one fail to realise the infinite potency that lies in devotion to the work which he has commanded himself to do. Love that into which one goes forward. Endow it with life; generate for it the magnetic atmosphere of hope, and belief, and conviction. One's personal power, carried into a new environment, shall produce external circumstances as beautiful as the power he brings to bear on them. It depends wholly on himself. If one is afraid of new conditions, if he dread the untried, it is really the fear of himself. But if he be strong in integrity of purpose, in singleness of aim, in that larger love and in the sweetness of spirit which is serenity and peace, he need feel no terror in going out into the unknown. . . . One need only trust. The force in to-day will rival and recreate the beautiful yesterday. If angels go out, the archangels may come in; and whether they do so or not depends wholly on oneself."

"That power is 'to him who power exerts' is as certain and unvarying a truth as any law of mathematics. The power that is within rushes to meet one from without. All that is one's own—that is, to-day—all that one has conquered of the invisible potencies by means of his own spiritual energy—all that power is his. No external change can deprive him of it. Nothing can lessen its force to create his outward life." Trials and perplexities, as a means of instruction, if we will acknowledge them to be such, the authoress considers a source of strength, by which we gain courage to meet and to master difficulties. "To keep one's foot firmly set in the way that leads upward, however dark and stormy it may be at the moment, is to conquer. All trial is in its very nature temporal; all joy is in its nature eternal. Legions of angelic powers wait on the soul and guide it to the Mount of Vision. After all, 'Each must live his life for himself; he must realise that that life lies between his own soul and God. Seek wisdom, seek understanding; but seek it at the Infinite Source. 'Trust thyself,' said Emerson, 'every heart vibrates to that string.'"

On the potent and undefinable quality of "charm" much is said that is arresting and true. "Charm is born and not made. It is temperamental. It is a gift, not an acquirement. . . . In so far as the individual possesses the ideal and artistic temperament, the poetic and imaginative as distinguished from that which is merely æsthetic and clever, so far he possesses the gift of charm. . . . The life of a certain type of individuals . . . is the life of penal servitude. Specific public duties have their place; but it is in the private life of the day in the more personal influences and immediate relations that the chief concern lies. To idealise this daily life, and to make it worth idealising, is the secret of this mysterious attraction." "Friendship" is a theme too time-worn for anything original to be said on it, one thinks, perhaps, and yet here before us stand out sentences embodying our own cherished ideas which, until now, we have not, possibly, seen in the light of day.

"Friends are discovered not made . . . but when discovered it is because of a predestined spiritual relation that compels recognition, and which transcends and dominates all temporary and external conditions or circumstances. Friendship of this order is as eternal as the spirit itself. It is part of spiritual identity, and simply cannot be destroyed. . . . Of course, this is the rarest order of friendship, which comes not only not more than once in a lifetime, perhaps not more than once among a hundred, or even a thousand lives." Probably in a friendship of this nature the process of idealisation must be very real. (Illusionment, cynics would call it.) But there is comfort in the following reflections:—"To idealise is not to *follow* a delusion, to mistake clay for alabaster; but it is to *see* more clearly, to discern that finer significance that he who runs may *not* read. It is only the exceptional nature that *can* be idealised—which is simply recognition for what is actually there, and not in the least a process of investing it with qualities it does not possess. It is the inner vision that sees 'the beauties hid from common sight.' Any friendship worthy of the name is a matter of magnetism and temperament. It can bear friction and jar, annoyance and pain, and still spring up again with renewed vitality. . . . People talk lightly of friendship when they do not know the meaning of the term; when they are not themselves of the stuff of which friends are made. . . . Friendship is in itself as fine an art as is music, or painting, or sculpture. . . . 'It is only the skilled and loving fingers of the artist who can bring forth what is noblest in tone, in touch, or in vision.' So with friendship; those who know, who see, who understand, are they who bring forth the hidden gold from the depths of a nature in sympathy with our own. 'Sympathetic companionship is the supreme luxury of life . . . and those who give it in any measure are comparatively few, and those who give it in perfect measure still more rare.'"

We will conclude our notice of this wise and understanding little book with some remarks on the distinction between a worldly woman and a woman of the world. "By the truer interpretation, a woman of the world, is not a 'worldly woman' at all. It requires far higher qualities than the selfishness and materialism that make a worldly woman to make one whose breadth of view, liberal sympathies, mental poise, and felicitous judgment fit her to be 'a woman of the world.'"

Wherever one opens "The Life Beautiful" there one finds brave, wise sayings. The subjects are not new, but the manner of treating them has a convincing freshness that is stimulating and inspiring.

### For Reading to the Sick.

THE SEASON OF LENT.

"THY Father sees in secret"—fast and prayer
And alms unknown to man, are all His own;
He treasures them in secret, storing them
Where nothing perishes. . . —*Isaac Williams.*

O my God, my Father! hear,
And help me to believe;
Weak and weary I draw near;
Thy child, O God, receive.
I so oft have gone astray;
To the perfect Guide I flee;
Thou wilt turn me not away,
Thy love is pledged to me.
*Hymns of the Spirit.*

Let us go then to-day to Him, who is the merciful receiver of all true penitent sinners—to Him, who willeth not that any should perish, but that all should be saved; go to Him, who not only calls us, but gives us the power to answer His call, presents us with His love, when we are yet a great way off . . . go to Him all through each day of Lent, crying, "Lord, save me;" and, believe me, we shall not be sent empty away.—*T. B. Dover.*

O child, hast thou fallen? arise, and go, with childlike trust, to thy Father, like the prodigal son, and humbly say, with heart and mouth, "Father, I have sinned against heaven, and before Thee, and am no more worthy to be called Thy son; make me as one of Thy hired servants." And what will thy heavenly Father do but what that father did in the parable? Assuredly He will not change His essence, which is love, for the sake of thy misdoings Is it not His own precious treasure, and a small thing with Him to forgive thee thy trespasses, if thou believe in Him? for His hand is not shortened that it cannot make thee fit to be saved.—*John Tauler.*

An occasional effort even of an ordinary holiness may accomplish great acts of sacrifice, or bear severe pressure of unwonted trial, specially if it be the subject of observation. But constant discipline in unnoticed ways, and the spirit's silent unselfishness, becoming the hidden habit of the life, give to it its true saintly beauty, and this is the result of care and lowly love in little things. Perfection is attained most readily by this constancy of religious faithfulness in all minor details of life, consecrating the daily efforts of self-forgetting love.—*T. T. Carter.*

Every hour comes with some little fagot of God's will fastened upon its back.—*F. W. Faber.*

Is any grieved or tired? Yea, by God's Will:
Surely God's Will alone is good and best:
O weary man, in weariness take rest,
O hungry man, by hunger feast thy fill.
Discern thy good beneath a mask of ill,
Or build of loneliness thy secret nest:
At noon take heart, being mindful of the west,
At night wake hope, for dawn advances still.

At night wake hope. Poor soul, in such sore need
Of wakening and of girding up anew,
Hast thou that hope which fainting doth pursue?
No saint but hath pursued and hath been faint;
Bid love wake hope, for both thy steps shall speed,
Still faint yet still pursuing, O thou saint.
*Christina Rossetti.*

### Notes and Queries.

**The Editor is always willing to answer in this column, without any fee, all reasonable questions, as soon as possible.**
**But the following rules must be carefully observed:—**
**1. Every communication must be accompanied by the name and address of the writer.**
**2. The question must always bear upon nursing, directly or indirectly.**
**If an answer is required by letter a fee of half-a-crown must be enclosed with the note containing the inquiry.**

*Treatment.*

(228) Will the Editor kindly tell "Mona" what is the general treatment for "peripheral neuritis."

The treatment will depend upon many things, the cause, the general condition of the patient. the part affected, the results produced, &c. It is entirely a matter for the physician in attendance.

*Midwife's Patient.*

(229) I am a midwife of great experience, holding the L.O.S. certificate, and have charge of a midwifery district. A patient delivered three months ago has developed pain in the right side. A local medical man, called in last week, sent the patient to our cottage hospital, has mentioned to my matron that something must have been wrong from time of delivery, and that now a serious operation must be performed. How could this latter trouble result from the confinement, or septic absorption have taken place without my knowledge? The patient had no rise of temperature, and I left her apparently well on the tenth day—viz. November 3rd. What operation could possibly be necessary under these circumstances? As I am greatly distressed about this case, and it is a most serious charge to have been made against me, I now solicit your kind opinion and advice on the subject.—*Puzzled.*

It is quite impossible to answer these questions or to say what is the matter with the patient. She may have a fibroid, or she may have an abscess, or even a lacerated perineum. No one who has not seen the patient can say what may be the matter with her.

*German Measles.*

(230) In connection with your article on Rubella or German Measles, will you kindly further inform me whether desquamation ever follows an attack, and, if so, at what period after the onset? Also, whether ulceration of the gums and lips ever precedes the illness? Your papers on infectious diseases are most valuable, because nearly all nurses in General Hospitals are carefully guarded from all contact with them, and have little opportunity of knowing much about a rash.—*E. F. G.*

Desquamation sometimes follows Rubella, often, in fact, and it may be copious, but it is generally of a fine and branny nature, not in the large sheets sometimes seen after scarlet fever. There is no particular day for its appearance.

*Dilemma.*

(231) I was booked to the patient with whom I am now from January 26th for five weeks. I am also engaged on March 22nd, or sooner if needed, to attend another lady. There is no prospect of the first confinement taking place for another fortnight, whilst the second threatens to be premature. What am I to do? My next patient and the surgeon say I am entitled to leave the first week in March.—*Perplexed.*

The matter is one for private arrangement. You must abide by your written agreement with both ladies, and each must provide a substitute in the event of the confinement taking place out of the appointed period. You should receive half fees for the case you are unable through no fault on your own part to attend.

*Nursing Home.*

(232) Will you kindly inform me if you know of any town or country place where two thoroughly-qualified nurses could work a home for convalescents or invalids?—*M. H.*

Reliable information can only be obtained from private sources, and the very greatest caution is necessary in negotiating enterprises of this kind.

*L.O.S.*

(233) I hold the L.O.S. certificate, but have no other training. What district nursing association would you recommend me to join?—*A. S.*

Your lack of training in general nursing renders you ineligible for district work.

*Book.*

(234) Can you tell me the name of a simple book concerning boys' ailments which would be useful to the matron of a public school?—*Z. B.*

The "Mother's Help and Guide to the Domestic Management of her Children," by Braidwood (price 2s., from Scientific Press), might be useful.

*Gynæcology.*

(235) Will you kindly tell me of a good up-to-date book on gynæcology for nurses?—*E. M. A.*

"Gynæcological Nursing," by G. A. Hawkins-Ambler, F.R.C.S. (price 1s., from Scientific Press), is very good.

*Training.*

(236) Will a year's certificate for general nursing and two or three months' maternity instruction be sufficient to qualify a nurse for private work? Would a doctor employ a nurse thus trained?—*Query.*

Yes. Medical men are glad to employ maternity nurses with some general training. But for general nursing, although one year is good, three years are better.

**Standard Books of Reference.**

"The Nursing Profession: How and Where to Train." 2s. net; post free, 2s. 4d.
"The Nurses' Dictionary of Medical Terms." 2s.
"Burdett's Series of Nursing Text-Books." 1s. each.
"A Handbook for Nurses." (Illustrated.) 5s.
"Nursing: Its Theory and Practice." New Edition. 3s. 6d.
"Helps in Sickness and to Health." Fifteenth Thousand.
"The Physiological Feeding of Infants." 1s.
"The Physiological Nursery Chart." 1s.; post free, 1s. 3d.
"Hospital Expenditure: The Commissariat." 2s. 6d.
All these are published by THE SCIENTIFIC PRESS, Ltd., and may be obtained through any bookseller or direct from the publishers, 28 and 29 Southampton Street, London, W.C.

